# A 6-month longitudinal study on worsening of Parkinson’s disease during the COVID-19 pandemic

**DOI:** 10.1038/s41531-022-00376-x

**Published:** 2022-08-31

**Authors:** Ali Shalash, Asmaa Helmy, Mohamed Salama, Ahmed Gaber, Mahmoud El-Belkimy, Eman Hamid

**Affiliations:** 1 Department of Neurology, Faculty of Medicine, Ain Shams University, Cairo, Egypt; 2 Institute of Global Health and Human Ecology (I-GHHE), the American University in Cairo, Cairo, Egypt; 3 Faculty of Medicine, Mansoura University, Mansoura, Egypt

**Keywords:** Parkinson's disease, Outcomes research

## Abstract

Further studies are required to investigate the impact of the COVID-19 pandemic on Parkinson’s disease (PD) progression. This study investigated the motor and non-motor progression of people with PD (PWP) at 6 months during the COVID-19 pandemic compared with that during the pre-pandemic period. Patients were recruited from Ain Shams University Hospitals, Cairo, in the period between April 2019 and December 2020. Fifty patients were included, of whom 17 and 33 patients were followed for 6 months before and during the pandemic, respectively. All patients were assessed at baseline and at 6 months using the MDS-UPDRS, Schwab and England scale (S&E), Hoehn and Yahr scale (H&Y), Berg Balance Scale, Timed Up and Go test (TUG), International Physical Activity Questionnaire, New Freezing of Gait Questionnaire, Non-Motor Symptoms Scale, and Beck Depression Inventory (BDI). Both groups were matched in age, gender, and disease characteristics. Patients followed during the pandemic showed more significant worsening of the total, part I and motor part of MDS-UPDRS, and balance scores (*p* < 0.001) than those followed during the pre-COVID-19 period. Gait (TUG), balance, and physical activity worsening were significantly correlated with baseline BDI, gait and balance scores, total and part I MDS-UPDRS scores, H&Y, and S&E OFF scores. Gait deterioration (TUG) was correlated with baseline physical activity (*r* = −0.510, *p* = 0.002). PWP showed worsening of motor and non-motor symptoms during the COVID-19 pandemic at the 6-month follow-up. Worsening of gait, balance, and physical activity was correlated with baseline motor and physical activity OFF scores.

## Introduction

Since the outbreak of the coronavirus disease 2019 (COVID-19) pandemic, individuals with Parkinson’s disease (PD) have been considered a vulnerable group to the effects of the COVID-19 pandemic, directly by infections with severe acute respiratory syndrome coronavirus 2 (SARS-CoV-2) and indirectly by pandemic-related restrictions, chronic stress, anxiety, physical inactivity, and compromised medical care. These effects included worsening of motor symptoms, higher mortality in advanced PD, worse anxiety and depression, impaired physical activity, and disruption of patients’ care^1–3^. Several recent studies have linked reduced physical activity and exercise to motor worsening during the pandemic, implying the significance of maintaining patients’ activity during restrictions^4,5^.

Moreover, it has been proposed that the COVID-19 pandemic may be followed by a higher incidence of neurodegenerative diseases; however, the evidence is insufficient to confirm that COVID-19 may trigger or accelerate neurodegeneration^6^. However, the impact of the COVID-19 pandemic and related measures on disease progression has not been explored. A recent retrospective study has reported a worsening of motor symptoms with a significant increase in motor disease progression during pandemic-related restrictions compared with that during the pre-pandemic period. The assessment was limited to ON-state and motor aspects^7^. Therefore, longitudinal studies are warranted to investigate the possibility of altered progression of motor and non-motor aspects of PD during the pandemic.

Accordingly, the current longitudinal study investigated the short-term motor and non-motor progression and related determinants of a cohort of people with PD (PWP) during the COVID-19 pandemic compared with the progression during the pre-pandemic period of another matched cohort.

## Results

Fifty patients were included, of whom 17 and 33 patients were followed for 6 months before and during the pandemic, respectively. Both groups were matched for demographic and clinical characteristics except for longer disease duration (*p* = 0.007) and lower rigidity ON scores (*p* = 0.01) for patients followed during the pandemic (Table 1). Physical activity ((International Physical Activity Questionnaire [IPAQ]) was non-significantly lower in patients during the pandemic. All patients did not report symptoms suggestive of COVID-19 infection. Cronbach’s alpha coefficient of the questionnaires ranged from 0.75 to 0.98, indicating a satisfactory internal consistency.

**Table 1:** Baseline characteristics of patients with PD who were followed before and during the COVID-19 pandemic.

		Patients followed before the COVID-19 period (*N* = 17)		Patients followed during the COVID-19 period (*N* = 33)		Mann–Whitney *U* test	
		Median/frequency	IQR/%	Median/frequency	IQR/%	*z*	*p*
Age^a^		55.71	6.65	56.65	10.64	−0.33^a^	0.74
Gender (male)^b^		13	76%	24	73%	0.08^b^	0.775
Years of education^a^		6.53	6.29	6.39	6.05	0.07^a^	0.941
AOO^a^		48.38	6.08	52.77	48.38	−1.54^a^	0.130
DOI^a^		6.38	3.33	4.10	6.38	2.79^a^	0.007*
Number of vascular risk factors		0	1	0	1	−0.49	0.619
Number of vascular risk factors^b^	0	10	59%	17	52%	2.53^b^	0.471
	1	4	24%	10	30%		
	2	3	18%	3	9%		
	3	0	0%	3	9%		
MDS-UPDRS total score OFF		79.50	75	79	49	−0.04	0.97
MDS-UPDRS total score ON		62.00	47	60.00	38.00	−0.30	0.77
MDS-UPDRS-I		15.50	15.50	18	8	−0.53	0.59
MDS-UPDRS-II		17.00	18.25	21	15	−0.32	0.75
MDS-UPDRS-III OFF		47.50	40	47.00	29.00	−0.51	0.61
MDS-UPDRS-III ON		30.00	24	29.00	20	−1.12	0.26
Rigidity OFF		9.50	9	8.00	5.00	−1.34	0.18
Rigidity ON		5.50	6	3.00	6	−2.49	0.01*
Bradykinesia OFF		16.50	19	15.00	10.00	−0.03	0.98
Bradykinesia ON		12.00	13	9.00	8.00	−0.86	0.39
PIGD OFF		7.00	12	7.00	9	−0.20	0.84
PIGD ON		5.00	9	6.00	5.00	−0.07	0.94
Axial OFF		11.00	12	13.00	10.00	−0.31	0.76
Axial ON		7.50	10	7.00	7	−0.19	0.85
Tremors OFF		15.00	11	11.00	13	−0.49	0.62
Tremor ON		9.00	8	6.00	9	−1.43	0.15
H&Y OFF		2.500	0.6	2.50	1.00	−0.30	0.77
H&Y ON		2.000	0.8	2.00	1.00	−0.14	0.89
Schwab and England ADL OFF		80.00	13	70.00	20.00	−0.55	0.58
Schwab and England ADL ON		85.00	10	80.00	20.00	−0.75	0.45
Motor complication total score		4.50	9	5.00	4.00	−0.45	0.65
TUG OFF		12.41	13.63	13	9	−0.57	0.57
TUG ON		11.60	5.03	10	5.50	−0.47	0.64
NFOG-Q OFF		12	22	0	21	−0.51	0.61
NFOG-Q ON		8	12	0	13	−0.84	0.40
BBS OFF		48	29	48	11	−0.06	0.95
BBS ON		53.50	12	53	6	−0.13	0.90
IPAQ		2,129.0	1,367.3	1,950.0	1,493.5	−1.08	0.28
LEDD		525	662.5	625	375	−0.52	0.61
MMSE		27.00	8	28.00	4.00	−0.30	0.76
NMSS total score		57	73	47	41	−0.26	0.80
BDI		18	12	19	15	−0.16	0.87
PDQ-39		35.41	41.59	46.35	26.85	−0.85	0.40

*AOO* age of onset, *DOI* duration of illness, *LEDD* levodopa equivalent daily dose, *MMSE* Mini-Mental State Examination, *NMSS* Non-Motor Symptoms scale, *PDQ-39* Parkinson’s Disease Questionnaire-39, *BDI* Beck Depression Inventory, *TUG* Timed Up and Go Test, *NFOG-Q* New Freezing of Gait, *BBS* Berg Balance Scale, *MDS-UPDRS* Movement Disorder Society–Unified Parkinson’s Disease Rating Scale, *PIGD* Postural Instability and Gait Disorder, *ADL* activities of daily living, *H&Y* Hoehn and Yahr, *IPAQ* International Physical Activity Questionnaire.

^a^T-test is used.

^b^Chi-square test is used.

*p-value is significant if <0.05.

**Corrected *p*-value is significant if ≤0.001 after Bonferroni’s adjustment.

### Disease progression before and during the COVID-19 pandemic

Patients followed before the pandemic showed a moderately significant progression at 6 months of MDS-UPDRS-I (Non-Motor Aspects of Experiences of Daily Living [nM-EDL]) (*p* = 0.044) and OFF-state motor scores (*p* = 0.047). The Schwab and England Activities of Daily Living scale (S&E) OFF- and ON-state scores showed a significant progression (*p* = 0.04 and 0.011, respectively) although not H&Y. Postural Instability and Gait Disorder (PIGD) ON and OFF-states, axial OFF-state, and New Freezing of Gait Questionnaire (NFOG-Q) OFF-state scores showed a moderately significant progression. The total non-motor symptoms scale (NMSS) and IPAQ showed significant worsening (*p* < 0.001) (Supplementary Table 1). The corrected *p*-value showed no significant changes, except for NMSS and IPAQ scores.

Patients followed during the COVID-19 pandemic showed a significant marked worsening at 6 months of MDS-UPDRS total and all subscores, S&E and Hoehn and Yahr (H&Y) (*p* < 0.001), PIGD, NFOG-Q, Timed Up and Go test (TUG), Berg Balance Scale (BBS) in OFF and ON states, total NMSS, IPAQ, and Mini-Mental State Examination (MMSE) (*p* < 0.001) (Supplementary Table 2).

Compared with the patients followed before the pandemic, those followed during the pandemic had greater significant worsening of the total and motor MDS-UPDRS OFF- and ON-state scores, MDS-UPDRS part I (*p* < 0.001), rigidity OFF (*p* = 0.04) and ON-state scores (*p* < 0.001), bradykinesia ON-state scores (*p* < 0.001), BBS OFF- and ON-state scores (*p* < 0.001) (significant after Bonferroni correction), axial OFF-state scores, and TUG (*p* = 0.01, non-significant after Bonferroni correction). Levodopa equivalent daily dosage (LEDD), MMSE, NMSS, IPAQ, and PDQ showed similar changes in both groups (Table 2).

**Table 2:** Comparison of 6-month disease progression before and during the COVID-19 pandemic.

Difference between the baseline and 6-month follow-up	Patients followed before the COVID-19 period (*N* = 17)		Patients followed during the COVID-19 period (*N* = 33)		Mann–Whitney *U* test		Percentage of change	
	Median	IQR	Median	IQR	*z*	*p*	Pre-COVID	Post-COVID
Δ MDS-UPDRS total score OFF	6	9.75	18	13.5	−3.29	<0.001**	8.94	20.97
Δ MDS-UPDRS total score ON	3	14.75	14	13	−3.88	<0.001**	6.06	27.59
Δ MDS-UPDRS-I	0	8	5	4	−3.87	<0.001**	0	26.67
Δ MDS-UPDRS-II	1.5	5.75	3	4	−0.83	0.4	22.22	14.28
Δ MDS-UPDRS-III OFF	4.5	6.75	8	7.5	−2.94	<0.001**	6.9	21.21
Δ MDS-UPDRS-III ON	−0.5	6.5	6	5.5	−3.51	<0.001**	−3.85	25
Δ Rigidity OFF	0	4.25	2	2	−2.09	0.04*	0	18.18
Δ Rigidity ON	−1	2.5	1	1.5	−3.63	<0.001**	−30	14.29
Δ Bradykinesia OFF	1.5	3.25	3	4	−1.93	0.05	9.38	22.22
Δ Bradykinesia ON	0	2.75	2	3	−2.83	<0.001**	0	20
Δ PIGD OFF	0.5	2.5	1	3	−0.82	0.41	5.26	21.43
Δ PIGD ON	0.5	2.25	1	2	−0.21	0.83	12.5	12.5
Δ Axial OFF	1	2.5	4	3	−2.54	0.01*	11.11	23.08
Δ Axial ON	1	2.25	2	2.5	−1.8	0.07	22.22	32.05
Δ Tremors OFF	0.5	5.5	1	2	−0.84	0.4	0	9.09
Δ Tremor ON	−0.5	5.5	0	2	−1.91	0.06	−17.65	0
Δ H&Y OFF	0	0.13	0.5	0.5	−1.55	0.12	0	20
Δ H&Y ON	0	0.13	0	0.5	−1.51	0.13	0	0
Δ Schwab and England ADL OFF	−10	10	−10	5	−1.37	0.17	−12.5	−12.5
Δ Schwab and England ADL ON	−5	10	−10	10	−0.89	0.37	0	−11.11
Δ Motor complication total score	0	4.5	1	1	−0.27	0.78	0	7.14
Δ TUG OFF	0.32	1.52	0.79	1.33	−2.62	0.01*	2.5	5.26
Δ TUG ON	0.5	1.37	0.5	0.8	−0.27	0.79	5	6.67
Δ NFOG-Q OFF	0	7.5	0	3.5	−0.29	0.77	4.2	8.04
Δ NFOG-Q ON	1.5	7	0	2.5	−0.5	0.62	12.5	15.97
Δ BBS OFF	0	4	−2	4	−2.94	<0.001**	1.82	−6.45
Δ BBS ON	0	2.25	−2	3	−2.86	<0.001**	0	−3.85
Δ IPAQ	−480.5	704.25	−377	392.75	−1.58	0.11	−30.47	−26.26
Δ MMSE	−1	2	0	1	−0.73	0.47	−1.67	0
Δ NMSS total score	8	8.5	9	6.5	−0.04	0.97	15.09	18.18
Δ PDQ-39	6.78	5.13	11.98	8.88	−1.34	0.18	20.63	27.76
Δ LEDD	0	300	0	337.5	−0.228	0.819		

Δ 6 m FU-baseline, *MMSE* Mini-Mental State Examination, *NMSS* Non-Motor Symptoms Scale, *PDQ-39* Parkinson’s Disease Questionnaire-39, *TUG* Timed Up and Go test, *NFOG-Q* New Freezing of Gait, *BBS* Berg Balance Scale, *MDS-UPDRS* Movement Disorder Society–Unified Parkinson’s Disease Rating Scale, *PIGD* Postural Instability and Gait Disorder, *ADL* activities of daily living, *H&Y* Hoehn and Yahr, *IPAQ* International Physical Activity Questionnaire, *LEDD* Levodopa equivalent daily dose.

*p-value is significant if <0.05.

**Corrected *p*-value is significant if ≤0.001 after Bonferroni’s adjustment.

### Correlations of disease progression during the pandemic

During the pandemic, the worsening of motor severity (H&Y) was correlated with disease duration (*r* = 0.500, *p* = 0.003), whereas motor complications (part IV) were directly correlated with baseline cognition (*p* = 0.02) and S&E (*p* = 0.03) and inversely correlated with depression and total and motor MDS-UPDRS scores (*p* = 0.04) (non-significant after Bonferroni correction); conversely, total and other MDS-UPDRS progression did not show significant correlations (Table 3).

**Table 3:** Correlations between disease progression during the COVID-19 pandemic and baseline characteristics.

		Δ MMSE	Δ NMSS	Δ TUG-OFF	Δ NFOG-Q OFF	Δ BBS OFF	Δ IPAQ	Δ MDS UPDRS Total OFF	Δ MDS UPDRS-I	Δ MDS UPDRS-II	Δ MDS UPDRS-III OFF	Δ H&Y OFF	Δ S&E OFF	Δ Motor complication total score
Age	Spearman	0.08	−0.15	0.27	−0.16	0.02	0.19	−0.11	−0.29	0.13	−0.07	0.1	−0.16	−0.25
	Sig.	0.65	0.41	0.13	0.36	0.91	0.29	0.56	0.1	0.47	0.7	0.59	0.37	0.17
Years of education	Spearman	0.41	0.01	−0.35	−0.07	0.27	−0.48	−0.04	0.1	0.23	−0.31	−0.02	−0.01	0.21
	Sig.	0.02	0.96	0.05	0.69	0.13	0.005*	0.81	0.6	0.19	0.08	0.91	0.97	0.24
AOO	Spearman	0.06	−0.21	0.27	−0.17	0.05	0.14	−0.14	−0.29	0.09	−0.11	−0.01	−0.16	−0.26
	Sig.	0.72	0.25	0.13	0.34	0.78	0.43	0.43	0.1	0.61	0.55	0.94	0.37	0.14
DOI	Spearman	0.16	0.19	0.19	0.07	−0.12	0.19	0.23	0.06	0.31	0.16	0.5	−0.21	0
	Sig.	0.37	0.29	0.28	0.72	0.49	0.29	0.2	0.74	0.08	0.36	0.003*	0.23	0.98
MMSE	Spearman	0.12	0.07	−0.33	−0.01	0.24	−0.52	−0.03	0.02	0.25	−0.23	−0.19	0.09	0.41
	Sig.	0.5	0.69	0.06	0.94	0.17	0.002**	0.85	0.93	0.16	0.2	0.28	0.63	0.02*
NMSS total score	Spearman	−0.12	−0.11	0.38	0.15	−0.35	0.3	0.04	−0.08	0.06	0.1	0.21	−0.11	−0.18
	Sig.	0.49	0.54	0.03*	0.4	0.05	0.09	0.8	0.65	0.73	0.57	0.24	0.55	0.31
BDI	Spearman	−0.16	−0.05	0.5	0.17	−0.46	0.51	0.09	0.01	−0.01	0.16	0.3	−0.22	−0.35
	Sig.	0.38	0.78	0.003*	0.35	0.007	0.002**	0.63	0.95	0.94	0.39	0.09	0.22	0.045*
TUG OFF	Spearman	−0.29	0.13	0.52	0.17	−0.45	0.51	0.26	0.05	0.16	0.28	0.18	−0.01	−0.35
	Sig.	0.1	0.46	0.002**	0.34	0.009*	0.002**	0.14	0.79	0.39	0.12	0.31	0.97	0.05
NFOG-Q OFF	Spearman	−0.33	0.45	0.51	0.24	−0.53	0.1	0.29	0.18	0.15	0.22	0.26	−0.04	−0.14
	Sig.	0.06	0.008*	0.003*	0.18	0.002**	0.57	0.1	0.32	0.41	0.23	0.14	0.85	0.45
BBS OFF	Spearman	0.26	−0.34	−0.5	−0.25	0.36	−0.34	−0.17	−0.01	−0.21	−0.11	−0.24	−0.02	0.21
	Sig.	0.14	0.06	0.003*	0.16	0.04*	0.05	0.36	0.95	0.23	0.53	0.17	0.92	0.24
MDS-UPDRS total score OFF	Spearman	−0.26	0.15	0.42	0.1	−0.35	0.52	0.07	0	−0.04	0.14	0.26	0	−0.36
	Sig.	0.15	0.41	0.02*	0.56	0.04*	0.002**	0.71	0.98	0.84	0.42	0.15	1	0.04*
MDS-UPDRS-I	Spearman	−0.1	−0.03	0.46	0.05	−0.38	0.39	−0.02	−0.24	0.09	0.06	0.14	−0.07	−0.16
	Sig.	0.59	0.87	0.007*	0.79	0.03*	0.02*	0.92	0.18	0.63	0.76	0.43	0.72	0.37
MDS-UPDRS-II	Spearman	−0.43	0.24	0.51	0.11	−0.36	0.51	0.09	0.09	−0.2	0.2	0.23	0.02	−0.34
	Sig.	0.01	0.19	0.003*	0.56	0.04	0.002**	0.63	0.6	0.27	0.28	0.2	0.9	0.06
MDS-UPDRS-III OFF	Spearman	−0.26	0.18	0.35	0.15	−0.31	0.43	0.09	0.04	0.02	0.12	0.25	−0.02	−0.36
	Sig.	0.14	0.32	0.05	0.41	0.08	0.01*	0.62	0.84	0.91	0.5	0.16	0.92	0.04*
PIGD OFF	Spearman	−0.37	0.36	0.61	0.24	−0.51	0.24	0.26	0.18	0.11	0.22	0.31	−0.08	−0.31
	Sig.	0.03*	0.04*	<0.001**	0.18	0.002**	0.17	0.15	0.32	0.53	0.22	0.07	0.66	0.08
Axial OFF	Spearman	−0.37	0.33	0.58	0.26	−0.42	0.39	0.24	0.15	0.11	0.22	0.31	−0.06	−0.29
	Sig.	0.04*	0.06	<0.001**	0.15	0.01*	0.02*	0.19	0.4	0.55	0.22	0.08	0.72	0.1
H&Y OFF	Spearman	−0.33	0.19	0.58	0.16	−0.36	0.47	0.21	0	0.22	0.19	0.21	−0.03	−0.33
	Sig.	0.06	0.28	<0.001**	0.39	0.04*	0.006*	0.24	0.98	0.23	0.29	0.24	0.85	0.06
Schwab and England ADL OFF	Spearman	0.34	−0.24	−0.54	−0.2	0.36	−0.49	−0.14	0	−0.05	−0.2	−0.23	−0.09	0.37
	Sig.	0.06	0.18	0.001**	0.28	0.04*	0.004*	0.44	0.99	0.78	0.27	0.19	0.62	0.03
Motor complication total score	Spearman	−0.12	0.16	0.37	0.35	−0.32	0.33	0.2	0.09	0.09	0.19	0.12	0.11	−0.31
	Sig.	0.51	0.37	0.04*	0.04*	0.07	0.06	0.27	0.61	0.63	0.29	0.52	0.53	0.08
IPAQ	Spearman	0.16	0	−0.51	−0.1	0.34	−0.63	−0.19	0	−0.14	−0.21	−0.3	0.05	0.27
	Sig.	0.37	1	0.002**	0.6	0.05	<0.001**	0.29	0.98	0.43	0.23	0.1	0.8	0.13

*AOO* age of onset, *DOI* duration of illness, *MMSE* Mini-Mental State Examination, *NMSS* Non-Motor Symptoms Scale, *TUG* Timed Up and Go test, *NFOG-Q* New Freezing of Gait, *BBS* Berg Balance Scale, *MDS-UPDRS* Movement Disorder Society–Unified Parkinson’s Disease Rating Scale, *PIGD* Postural Instability and Gait Disorder, *ADL* activities of daily living, *H&Y* Hoehn and Yahr, *IPAQ* International Physical Activity Questionnaire.

*p-value is significant if <0.05.

**Corrected *p*-value is significant if ≤0.002 after Bonferroni’s adjustment.

Cognitive worsening was correlated with years of education, baseline MDS-UPDRS part II (*r* = −0.430, *p* = 0.01), PIGD (*r* = −0.370, *p* = 0.03), and axial scores (*r* = −0.370, *p* = 0.03). Total NMSS worsening was correlated with baseline NFOG-Q (*r* = 0.450, *p* = 0.01) and PIGD scores (*r* = 0.360, *p* = 0.04) (non-significant after Bonferroni correction). TUG (OFF), BBS (OFF), and IPAQ worsening were significantly correlated with baseline Beck Depression Inventory (BDI), gait (TUG) and balance (BBS) OFF scores, total and part I MDS-UPDRS scores, and H&Y and S&E scores. After Bonferroni correction, a significant correlation was noted between TUG and baseline OFF-state TUG, PIGD, axial, H&Y, S&E, and IPAQ scores; BBS and baseline OFF PIGD and NFOG-Q; and IPAQ and baseline MMSE, MDS-UPDRS-total and part II-OFF, BDI, TUG-OFF, and IPAQ scores (*p* ≤ 0.002) (Table 3).

On comparison between the two groups regarding the assessment scale scores after 6 months, significant worsening was noted in MDS-UPDRS-I in the patients followed during the COVID-19 period (*p* = 0.003) (Supplementary Table 3).

## Discussion

Several cross-sectional studies have constantly described the worsening of motor and non-motor symptoms during the COVID-19 pandemic. However, its impact on disease progression was not adequately investigated. Distinctively, this longitudinal study explored disease progression during the pandemic and showed worsening of motor and non-motor symptoms over a 6-month follow-up during the pandemic compared with that during the pre-pandemic period. Gait, balance, and physical activity worsening were correlated with baseline motor and physical activity scores. The current study showed the possibility of deleterious effects of pandemic lockdown on disease progression in PWP.

The current findings are consistent with those of previous cross-sectional studies. An Indian study showed worsening of motor symptoms, especially bradykinesia in 69.2% of cases during the COVID-19 pandemic, followed by tremor, rigidity, and gait freezing^8^. Additionally, PWP reported worsening in mental health, quality of life, and physical inactivity during this pandemic^5^. These indirect effects of COVID-19 are more confirmed and may be more common and more harmful than the direct effects of viral infections^1,9,10^. The worsening of motor and non-motor symptoms has been attributed to stress, physical inactivity, pharmacodynamic effects, dramatic changes in routine, and social isolation^11,12^. The impact of stress on PD progression has been previously investigated and proven to negatively affect the course of the disease^1,2,4^.

Conversely, the impact on disease progression is not well investigated. However, a recent study by Ineichen et al. has reported increased motor disease progression during pandemic-related restrictions compared with that before the COVID-19 pandemic, which is consistent with the current study^7^. Similar to symptom worsening, more deterioration during the pandemic could be explained by stress, physical inactivity, and social isolation.

PWP are more vulnerable to recent stressors, which is attributed to more dopamine depletion and consequently reduced coping mechanisms for stress^13^. Moreover, chronic stress may induce oxidative damage to the cell membrane, as well as inflammatory and regulatory T-cell dysfunction, leading to a possible increase in midbrain dopaminergic neuron loss and motor symptom worsening^14^. Additionally, chronic stress accelerates dopaminergic cell loss in animal PD models and exacerbates the neuropathological changes^15^. The accompanying microglial activation and oxidative stress may mechanistically justify the stress-induced neurodegeneration in PD^16^.

Remarkably, gait (TUG), balance, and physical activity were markedly worsened during the pandemic, which was related to baseline motor and physical activity scores. Consistently, a recent study, which assessed 12 patients before and 2 months after lockdown, has shown moderate gait, falls, and balance worsening although not freezing of gait, despite being contacted by a multidisciplinary team^17^.

Approximately 29% of patients with PD are less physically active than the normal population, which are predicated by disease severity, gait, and impairment of activities of daily living^18^. Increased physical activity and exercise have a positive effect on motor and non-motor symptoms and probably PD disease progression^19^. Moreover, exercise may improve the progression of PD manifestations and enhance motor and cognitive circuit-related neuroplasticity^20–22^.

Despite there being no reported cases of SARS-CoV-2 infection, its direct effect on disease progression due to subclinical infection could not be totally excluded. An Italian study reported motor and non-motor deterioration that was attributed to infection and drug pharmacodynamic-related mechanisms. A quarter of patients with PD and COVID-19 had mild symptoms and recovered without treatment^23^.

SARS-CoV-2-related neurodegenerations, especially nigrostriatal degenerations, have been proposed owing to the possibility of neuro-invasion via the olfactory bulb, Angiotensin-Converting Enzyme 2 (ACE2) receptor expression by dopaminergic neurons, associated inflammatory and vascular changes that overlap with PD pathogenesis, and progression in addition to reported cases of SARS-CoV-2-related parkinsonism with dopamine transporter imaging abnormalities^1,24^. However, the impact of COVID-19 on neurodegeneration, including the development or increased progression of PD, has not yet been confirmed.

The strengths of the current study include the comprehensive assessment in the OFF and ON states and follow-up of patients with mild-to-moderate PD before and during the pandemic, with a comprehensive assessment of motor and non-motor symptoms and matched baseline characteristics of patients in both groups.

The current study has some limitations. The small number and short follow-up period, as well as the lack of confirmation or exclusion of SARS-CoV-2 infection using laboratory tests during assessments, are considered its limitations. Furthermore, our cohort included patients with mild-to-moderate PD who should be considered in the interpretation of our findings.

This study confirms the impact of the COVID-19 pandemic on the motor and non-motor symptoms of PD and demonstrates another possible effect, which is the short-term worsening of disease progression, implying the significance of managing related factors, including anxiety, chronic stress, and physical inactivity, and considering this effect in interpreting longitudinal studies during the pandemic.

## Methods

This is a retrospective analysis of the data from our longitudinal study (PDPRO-EGY, clinicalTrial.gov, NCT04062279) that investigated the progression of Egyptian patients with PD. Patients were recruited from the movement disorders outpatient clinic of Ain Shams University Hospitals (Cairo, Egypt) in the period between April 2019 and December 2020. Patients with PD who were diagnosed using the International Parkinson and Movement Disorders Society (MDS) diagnostic criteria^25^ and completed both baseline and 6-month follow-up assessments before or during the pandemic period were included and compared. Patients who completed baseline and follow-up assessments during the pandemic were included as the “one group” (from March to December 2020), whereas those who completed the follow-up before March 2020 (before lockdown procedures in Egypt) were included as the “control group.”

Patients excluded include patients with atypical or acquired parkinsonism, those who did not complete the follow-up assessment, those who were assessed in the pre-pandemic (baseline) and during the pandemic (6-month follow-up) periods, and those who underwent functional brain surgery (before or during the follow-up period).

### Sampling and sample size

The sample size was calculated using an online calculator (https://www.calculator.net/sample-size-calculator.html), where for a 95% confidence level and a margin of error 5, the minimal sample size was estimated to be 16 patients for each group.

### Ethical considerations

All participants provided written informed consent. The study was approved by the ethical committee of the Faculty of Medicine of Ain Shams University according to the Declaration of Helsinki.

### Data collection

All patients were evaluated at baseline and at 6-month follow-up using the total and different parts of the MDS-UPDRS (parts include part I [nM-EDL], part II [motor aspects of daily living], part III [motor examination], and part IV [motor complications]), H&Y for disease severity, S&E for activities of daily living^26^, NFOG-Q^27^ for gait freezing, BBS^28^ for balance assessment, and TUG^29^ for gait and mobility assessment during the OFF and ON states. Other scales included the IPAQ-SF^30^ for physical activities, NMSS^31^ for non-motor symptoms evaluation, Arabic version of BDI^32^ for depression, Arabic version of PD questionnaire 39^33^ for quality of life, and MMSE^34^ for cognition. All patients have been evaluated in person by a trained physician. LEDD was calculated at baseline and follow-up as the sum of the daily dose of all dopaminergic agents^35^.

### Statistical analysis

Data analysis was performed using IBM SPSS software package version 25.0 (IBM Corp., Armonk, NY). Qualitative data were described as frequencies and percentages and compared using the chi-square test, whereas quantitative data were presented as medians and interquartile ranges or means ± standard deviations and compared using either the Mann–Whitney *U* test or Student’s *t* test according to the distribution of the data, respectively. The Wilcoxon signed-rank test was used to compare baseline and follow-up data within each group. The Spearman correlation coefficient was used to evaluate the correlation between different variables. The significance was set at *p* < 0.05. Bonferroni correction was performed for the multiple comparisons and correlations, and an adjusted p-value was used. Cronbach’s α coefficient was used as a measure of the internal consistency of used questionnaires.

## Supplementary information

Supplemntary Tables

## Data Availability

The datasets generated during the current study will be made available from the corresponding author upon request.
