# Supplementary material for: A 6-month longitudinal study on worsening of Parkinson’s disease during the COVID-19 pandemic
Source: NPJ Parkinsons Dis. 2022 Aug 31;8:111. doi: 10.1038/s41531-022-00376-x (PMC9428872; doi:10.1038/s41531-022-00376-x)
Supplement: Supplementary file 1 — Supplemntary Tables [file 41531_2022_376_MOESM1_ESM.pdf]

Supplementary Tables

Supplementary Table 1: comparison of baseline and 6-months follow-up of patients with PD in pre COVID -19 pandemic:

	Baseline		6 months Follow-up		Wilcoxon Signed Ranks Test	
	Median	IQR (range	Median	IQR (range	z	p
Total MDS-UPDRS OFF	15.50	15.50	13.50	9.50	-0.04	0.972
Total MDS-UPDRS ON	17	18.25	22.50	13.00	-1.53	0.126
MDS-UPDRS -I	79.50	75	85	45	-2.01	0.044*
MDS-UPDRS -II	62	47	63.50	36	-0.31	0.757
MDS-UPDRS-III OFF	47.50	40	53	31	-1.99	0.047*
MDS-UPDRS-III ON	30	24	29.50	27	-0.46	0.649
Rigidity-OFF	9.50	9	10	8	-0.54	0.588
Rigidity-ON	5.50	6	6.50	5	-1.73	0.083
Bradykinesia-OFF	16.50	19	16.50	11	-0.91	0.362
Bradykinesia-ON	12	13	12.00	11	-0.54	0.591
PIGD-OFF	7	12	7	14	-2.02	0.044*
PIGD-ON	5	9	6.50	7	-1.98	0.048*
Axial-OFF	11	12	14	11	-1.978	0.048*
Axial-ON	7.50	10	8	7	-1.706	0.088
Tremors-OFF	1	11	15	10	-.915	0.360
Tremor-ON	9	8	8.50	8	-.571	0.568
H&Y-OFF	2.50	.6	2.50	.9	-1.890	0.059
H&Y -ON	2	.8	2	1.1	-1.633	0.102
Schwab and England ADL OFF	80	13	70	25	-2.887	0.004*
Schwab and England ADL ON	85	10	80	23	-2.530	0.011*
Motor complication total score	4.50	9	6	6	-1.752	0.080
MMSE	27	8	26	7	-1.88	0.061
Total NMSS	57	73	60.50	72	-3.58	<0.001**
TUG OFF	12.41	13.63	13.25	28.25	-1.61	0.107
TUG ON	11.60	5.03	12.40	6.63	-1.87	0.061
NFOG-Q OFF	11	20	15.00	27	-2.03	0.042*
NFOG-Q ON	4	12	7.00	18	-1.88	0.061
BBS OFF	48	29	48.50	20	-0.36	0.716
BBS ON	53.50	12	52.50	11	-1.25	0.212
IPAQ	2129	1367.3	1571	914.0	-3.622	<0.001**
LEDD	525	662.5	550	524.5	-0.105	0.916

MMSE; Mini Mental State Examination, NMSS ; Non-Motor Symptoms Scale,TUG; time up and go test, NFOG-Q ; New Freezing Of Gait, BBS ; Berg Balance Scale, MDS-UPDRS; Movement Disorder Society –

Unified Parkinson's Disease Rating Scale, PIGD; Postural Instability And Gait Disorder, S&E-ADL; Schwab and England-Activities Of Daily Living, H&Y; Hoehn and Yahr, IPAQ ; International Physical Activity Questionnaire, LEDD; Levodopa equivalent daily dose

*p value is significant if <0.05
**Correccted p value is significant if ≤ 0.001 after Bonferroni's adjustment

Supplementary Table 2: comparison of baseline and 6-months follow-up of patients with PD followed during COVID-19 pandemic:

	Baseline		6 months Follow-up		Wilcoxon Signed Ranks Test	
	Median	IQR	Median	IQR	Z	p
Total MDS-UPDRS OFF	18	8	22	7.5	-4.780	<0.001**
Total MDS-UPDRS ON	21	15	25	14	-5.013	<0.001**
MDS-UPDRS -I	79	49	102	48	-5.01	<0.001**
MDS-UPDRS -II	60	38	77	38	-5.00	<0.001**
MDS-UPDRS-III OFF	47	29	60	29.50	-5.02	<0.001**
MDS-UPDRS-III ON	29	20	32	23.50	-4.85	<0.001**
Rigidity-OFF	8	5	10	6	-4.35	<0.001**
Rigidity-ON	3	6	6	5.50	-3.91	<0.001**
Bradykinesia-OFF	15	10	21	10	-4.37	<0.001**
Bradykinesia-ON	9	8	11	9	-3.45	<0.001**
PIGD-OFF	7	9	10	9.50	-3.70	<0.001**
PIGD-ON	6	5	7	7	-3.31	<0.001**
Axial-OFF	13	10	17	9.50	-4.81	<0.001**
Axial-ON	7	7	10	8	-4.81	<0.001**
Tremors-OFF	11	13	13	13.50	-3.57	<0.001**
Tremor-ON	6	9	5	8	-2.28	0.02*
H&Y-OFF	2.50	1	3	1.50	-3.74	<0.001**
H&Y -ON	2	1	2.50	1	-3.74	<0.001**
S&E- ADL OFF	70	20	70	20	-4.91	<0.001**
S&E- ADL ON	80	20	80	20	-4.49	<0.001**
Total motor complications	5	4	6	4	-3.57	<0.001**
MMSE	28	4	27	4	-2.43	0.02*
NMSS total score	47	41	62	38.50	-4.92	<0.001**
TUG OFF	1	9	15.50	9.63	-5.02	<0.001**
TUG ON	10	5.50	11.80	5.65	-4.84	<0.001**
NFOG-Q OFF	0	21	16	26.50	-3.42	<0.001**
NFOG-Q ON	0	13	0	16	-3.07	<0.001**
BBS OFF	48	11	45	17	-4.47	<0.001**
BBS ON	53	6	52	10	-4.47	<0.001**
IPAQ	1950	1493.50	1371	1268.3	-4.86	<0.001**
LEDD	625	375	525	375	-0.227	0.820

MMSE; Mini Mental State Examination, NMSS ; Non-Motor Symptoms Scale,TUG; time up and go test, NFOG-Q ; New Freezing Of Gait, BBS ; Berg Balance Scale, MDS-UPDRS; Movement Disorder Society –

Unified Parkinson's Disease Rating Scale, PIGD; Postural Instability and Gait Disorder, S&E-ADL; Schwab and England-Activities Of Daily Living, H&Y; Hoehn and Yahr, IPAQ; International Physical Activity Questionnaire, LEDD; Levodopa equivalent daily dose

*p value is significant if <0.05
**Correccted p value is significant if ≤ 0.001 after Bonferroni's adjustment

Supplementary Table 3: Comparison of follow up at 6 months in pre and during COVID-19 pandemic

	Patients followed in Pre COVID-19 period (No=17)		Patients followed during COVID-19 period (No =33)		MANN WHITNEY U TEST	
	Median	IQR	Median	IQR	z	p
MDS-UPDRS total score OFF	85	49	102	48	-1.270	0.204
MDS-UPDRS total score ON	60	36	77	38	-1.321	0.186
MDS-UPDRS -I	14	9.50	22	7.50	-2.946	0.003*
MDS-UPDRS -II	23	12.50	25	14	-1.046	0.296
MDS-UPDRS-III OFF	51	34	60	30	-0.410	0.682
MDS-UPDRS-III ON	29	27	32	24	-0.287	0.774
Rigidity-OFF	10	7	10	6	-0.432	0.666
Rigidity-ON	6	5	6	6	-0.258	0.796
Bradykinesia-OFF	17	11	21	10	-0.833	0.405
Bradykinesia-ON	12	12	11	9	-0.586	0.558
PIGD-OFF	7	12	10	10	-0.092	0.926
PIGD-ON	6	7	7	7	-0.411	0.681
Axial-OFF	14	11	17	10	-0.770	0.441
Axial-ON	8	7	10	8	-0.493	0.622
Tremors-OFF	15	11	13	14	-0.369	0.712
Tremor-ON	8	10	5	8	-0.710	0.478
H&Y-OFF	2.5	1.0	3	1.5	-1.111	0.267
H&Y -ON	2	1.5	2.50	1.0	-0.582	0.560
Schwab and England ADL OFF	70	30	70	20	-1.057	0.291
Schwab and England ADL ON	80	20	80	20	-1.134	0.257
Motor complication total score	6	5	6	4	-0.846	0.398
TUG OFF	13.50	30.25	15.50	9.63	-0.635	0.525
TUG ON	12.30	6.05	11.80	5.65	-0.420	0.674
NFOG-Q OFF	15	26	16	27	-0.348	0.728
NFOG-Q ON	7	19	0	16	-0.652	0.514
BBS OFF	49	18	45	17	-0.892	0.372
BBS ON	53	12	52	10	-0.720	0.471

IPAQ	1472	894	1371	1268.3	-0.522	0.601
LEDD	550	524.5	525	375	-0.69	0.491
MMSE	27	5	27	4	-1.001	0.317
NMSS total score	65	69	62	39	-0.184	0.854
BDI	27	16	25	17	-1.118	0.264
PDQ-39	42.66	28.75	51.51	27.92	-1.178	0.239

MMSE;mini mental state examination, NMSS ; non-motor symptoms scale, PDQ_39; Parkinson's disease questionnaire-39, BDI; beck depression inventory, TUG; time up and go test, NFOG-Q; new freezing of gait, BBS; berg balance scale, MDS-UPDRS; movement disorder society – unified Parkinson's disease rating scale, PIGD; postural instability and gait disorder, ADL; activities of daily living, H&Y ;Hoehn and Yahr, IPAQ; international physical activity questionnaire,

*p value is significant if <0.05
**Correccted p value is significant if ≤ 0.001 after Bonferroni's adjustment
